# Association of GDF15 levels with body mass index and endocrine status in β‐thalassaemia

**DOI:** 10.1111/cen.14897

**Published:** 2023-02-27

**Authors:** Yanislava Karusheva, Clive J. Petry, Nirmani Yasara, Dulani Kottahachchi, Anuja Premawardhena, Peter Barker, Keith Burling, Naveed Sattar, Paul Welsh, Sachith Mettananda, Stephen O'Rahilly

**Affiliations:** ^1^ MRC Metabolic Diseases Unit, Wellcome‐MRC Institute of Metabolic Science University of Cambridge Cambridge UK; ^2^ NIHR Cambridge Biomedical Research Centre Cambridge UK; ^3^ Department of Paediatrics, Faculty of Medicine University of Kelaniya Ragama Sri Lanka; ^4^ Department of Physiology, Faculty of Medicine University of Kelaniya Ragama Sri Lanka; ^5^ Colombo North Teaching Hospital Ragama Sri Lanka; ^6^ Department of Medicine, Faculty of Medicine University of Kelaniya Ragama Sri Lanka; ^7^ Core Biochemical Assay Laboratory Cambridge University Hospitals NHS Foundation Trust Cambridge UK; ^8^ University of Glasgow, School of Cardiovascular and Metabolic Health Glasgow UK

**Keywords:** beta‐thalassaemia, body mass index, GDF15, IGF 1

## Abstract

**Objective:**

GDF15 has emerged as a stress‐induced hormone, acting on the brain to reduce food intake and body weight while affecting neuroendocrine function. Very high GDF15 levels are found in thalassaemia, where growth, energy balance and neuroendocrine function are impaired. We examined the relationships between GDF15 and anthropometric measures and endocrine status in β‐thalassaemia.

**Design:**

Cross sectional study.

**Patients:**

All β‐thalassaemia patients attending the thalassaemia unit of Colombo North Teaching Hospital for blood transfusions.

**Measurements:**

Anthropometric data, appetite scores, circulating GDF15, IGF, thyroid and reproductive hormone levels in 103 β‐thalassaemia patients were obtained.

**Results:**

GDF15 levels were markedly elevated in thalassaemia patients (24.2‐fold with β‐thalassaemia major compared with healthy controls). Among patients with β‐thalassaemia major, the relationship between GDF15 and body mass index (BMI) was curvilinear with all individuals with GDF15 levels above 24,000 pg/mL having a BMI below 20 kg/m^2^. After adjustment for BMI, age and Tanner stage, serum IGF1 concentrations correlated negatively with GDF15 in all thalassaemia patients (*β* = −.027, *p* = .02). We found a significant positive relationship between GDF15 and gonadotropin (in both sexes) and testosterone (in males).

**Conclusions:**

GDF15 levels were markedly elevated in patients with β‐thalassaemia and its association with BMI is consistent with the known effect of GDF15 to reduce body weight. The inverse association between GDF15 with IGF1 levels may reflect a neuroendocrine impact of GDF15 or an indirect effect via impaired nutritional state. The positive association with testosterone in males and gonadotropins in both sexes, was surprising and should prompt further GDF15 studies on the hypothalamic pituitary gonadal axis.

## 1 INTRODUCTION

GDF15 is an endocrine signal of cellular stress.^1^ Its levels are elevated in a wide range of chronic human diseases where they frequently correlate with disease severity and adverse outcomes.^2^, ^3^, ^4^ GDF15 acts on receptors in the brain to elicit a range of illness behaviours and a neuroendocrine stress response.^5^ Patients with thalassaemia have previously been reported to have extremely high levels of GDF15, which is believed to be derived from their expanded mass of abnormal red cell precursors.^6^, ^7^, ^8^ GDF15 expression is increased in cells subject to an unfolded protein response^9^ which is a prominent feature of thalassaemia erythroblasts.^6^ Other studies have suggested a role for intracellular iron in the control of GDF15 expression.^10^ Despite regular transfusion and the use of iron chelating agents, patients with thalassaemia still frequently suffer from problems of growth and development, endocrine dysfunction and poor quality of life.^11^, ^12^, ^13^ We have undertaken studies in patients with thalassaemia to examine the relationship between levels of GDF15, anthropometric variables and endocrine parameters.

## 2 MATERIALS AND METHODS

### 2.1 Study participants

A cross sectional study was conducted at the Adolescent and Adult Thalassaemia Centre of the Colombo North Teaching Hospital, Ragama, Sri Lanka from January to March 2021. All patients with β‐thalassaemia attending for blood transfusions during the study period were recruited into the study after obtaining informed written consent. A total of 103 patients with β‐thalassaemia [78 with β‐thalassaemia major, 18 with haemoglobin E (HbE) β‐thalassaemia and 7 with β‐thalassaemia intermedia] were recruited. The diagnosis of β‐thalassaemia had been confirmed by haemoglobin subtype quantification by high performance liquid chromatography. We also recruited 5 carriers of β‐thalassaemia trait (randomly selected from parents of patients with thalassaemia) and 5 non‐thalassaemia controls (randomly selected from unaffected family members of patients) for the study. The ethical approval was obtained from the Ethics Review Committee of the Faculty of Medicine, University of Kelaniya, Sri Lanka (Ref. P/228/11/20l9) and performed in accordance to the declaration of Helsinki.

### 2.2 Data collection procedure

After recruitment, basic sociodemographic and clinical data were gathered using a data collection form by interviewing patients and perusing clinical records. Height and weight were measured using calibrated instruments and pubertal stage was assessed using Tanner staging. Nonspecific symptoms related to food intake and lifestyle were gathered using questions that were answered ‘yes’ or ‘no’. Pretransfusion venous blood samples were collected during morning hours (at 9.00 am where possible) and freshly separated plasma were stored and shipped frozen to Cambridge, United Kingdom for the measurement of GDF15, IGF1, oestradiol and testosterone. Haematological and other measurements were done in Sri Lanka. All participants were afebrile and free from signs of infection or inflammation at the time of blood sampling.

### 2.3 Analytical methods

Samples were analysed for GDF‐15 using the Roche Elecsys® e411 GDF‐15 Cobas electrochemiluminescent immunoassay (Roche Diagnostics). The assay range is 400−20,000 pg/mL, with samples that were initially above the measuring range being diluted in Roche ‘Multiassay Diluent’. Assays were calibrated and quality controlled using the manufacturer's reagents. Over two levels of controls, the coefficient of variation (CoV) of both controls was <5%.

IGF‐1 levels were measured using the DiaSorin Liaison® XL IGF‐1 one‐step sandwich chemiluminescence immunoassay after prior separation of IGF‐1 from binding proteins (DiaSorin). The assay range is 1.3−130 nmol/L. The assay is referenced to the first WHO International Standard for IGF‐1 NIBSC code 02/254. The CoV of the IGF‐1 assay is 9.6% at 10.4 nmol/L, 7.1% at 24.3 nmol/L, 5.6% at 41.2 nmol/L.

Assays were calibrated and quality controlled using the manufacturer's reagents. The measurement of serum oestradiol (CoV: 4.5% at 451 pmol/L, 4.2% at 1589 pmol/L) and testosterone (CoV: 5.0% at 5.97 nmol/L, 3.5% at 21.27 nmol/L) were done using direct competitive chemiluminescence immunoassays on the on the DiaSorin Liaison® XL (DiaSorin). Reagents, calibrators and quality controls were supplied by DiaSorin.

Full blood counts were performed using Beckman Coulter LH 500 automated full blood count analyser. Serum TSH, free thyroxine, LH, FSH and cortisol were measured using Tosoh Bioscience® AIA‐360 automated chemiluminescence immunoassay analyser in a clinically accredited laboratory.

### 2.4 Comparative statistical analyses

Continuous variables were analysed using linear regression (transforming data before analysis where necessary so that the residuals were normally distributed). Confounders [particularly sex, age and body mass index (BMI)] were added to statistical models as appropriate. After visual inspection of data using scatter diagrams, in certain cases quadratic associations were tested by adding squared independent variables (e.g., BMI) into linear regression models. Missing data were treated by pairwise deletion. Data are shown as geometric means (95% confidence intervals) unless stated otherwise. Statistical analyses were performed using Stata (version 13.1; Stata Corp.; from Timberlake Consultants Ltd.).

## 3 RESULTS

### 3.1 Background characteristics of study participants

Of the 103 patients with thalassaemia, 59 (57.3%) were males. The mean age of β‐thalassaemia patients was 24.6 (±9.5) years and that of β‐thalassaemia carriers and non‐thalassaemia controls were 40.4 (±1.5) and 34.2 (±8.3) years, respectively. The clinical characteristics of patients with different subtypes of β‐thalassaemia are shown in Table 1. The nonspecific symptoms related to food intake and lifestyle among patients with thalassaemia are given in Supporting Information: table 1.

**Table 1:** Clinical characteristics of b‐thalassaemia patients.

Characteristic	Subtypes of β‐thalassaemia			All patients with β‐thalassaemia (*N* = 103)
	β‐thalassaemia major (*N* = 78)	HbE β‐thalassaemia (*N* = 18)	β‐thalassaemia intermedia (*N* = 7)	
Sex				
Male	49 (62.8%)	8 (44.4%)	2 (28.6%)	59 (57.3%)
Female	29 (37.2%)	10 (55.6%)	5 (71.4%)	44 (42.7%)
Age (years)	22.3 (±6.0)	27.7 (±12.5)	43.1 (±12.0)	24.6 (±9.5)
Age at diagnosis (months)	7.8 (±6.7)	86.0 (±148)	257 (±188)	38 (±101)
Annual transfusion requirement (mL/kg/year)	184 (±48)	128 (±61)	103 (±74)	169 (±59)
Pretransfusion haemoglobin (g/dL)	8.72 (±0.85)	6.36 (±1.81)	6.45 (±1.46)	8.2 (1.4)
Serum ferritin (ng/mL)	2207 (±1735)	1476 (±1065)	2125 (±1986)	2074 (±1665)
Disease complications				
Facial changes	5 (6.4%)	0 (0%)	0 (0%)	5 (4.9%)
Hepatomegaly	33 (42.3%)	15 (83.3%)	5 (71.4%)	53 (51.5%)
Splenectomized	15 (19.2%)	6 (33.3%)	1 (14.3%)	22 (21.4%)
Transfusion reactions	44 (56.4%)	9 (50.0%)	2 (28.6%)	55 (53.4%)
Hypothyroidism	11 (14.1%)	1 (5.6%)	3 (42.9%)	15 (14.6%)
Hypoparathyroidism	13 (16.7%)	3 (16.7%)	0 (0%)	16 (15.5%)
Diabetes	13 (16.7%)	1 (5.6%)	2 (28.6%)	16 (15.5%)
Osteoporosis	7 (9.0%)	1 (5.6%)	2 (28.6%)	10 (9.7%)

*Note*: Data are shown as *n* (%) or mean (±SD).

### 3.2 GDF15 levels in patients with β‐thalassaemia

**Figure 1:**
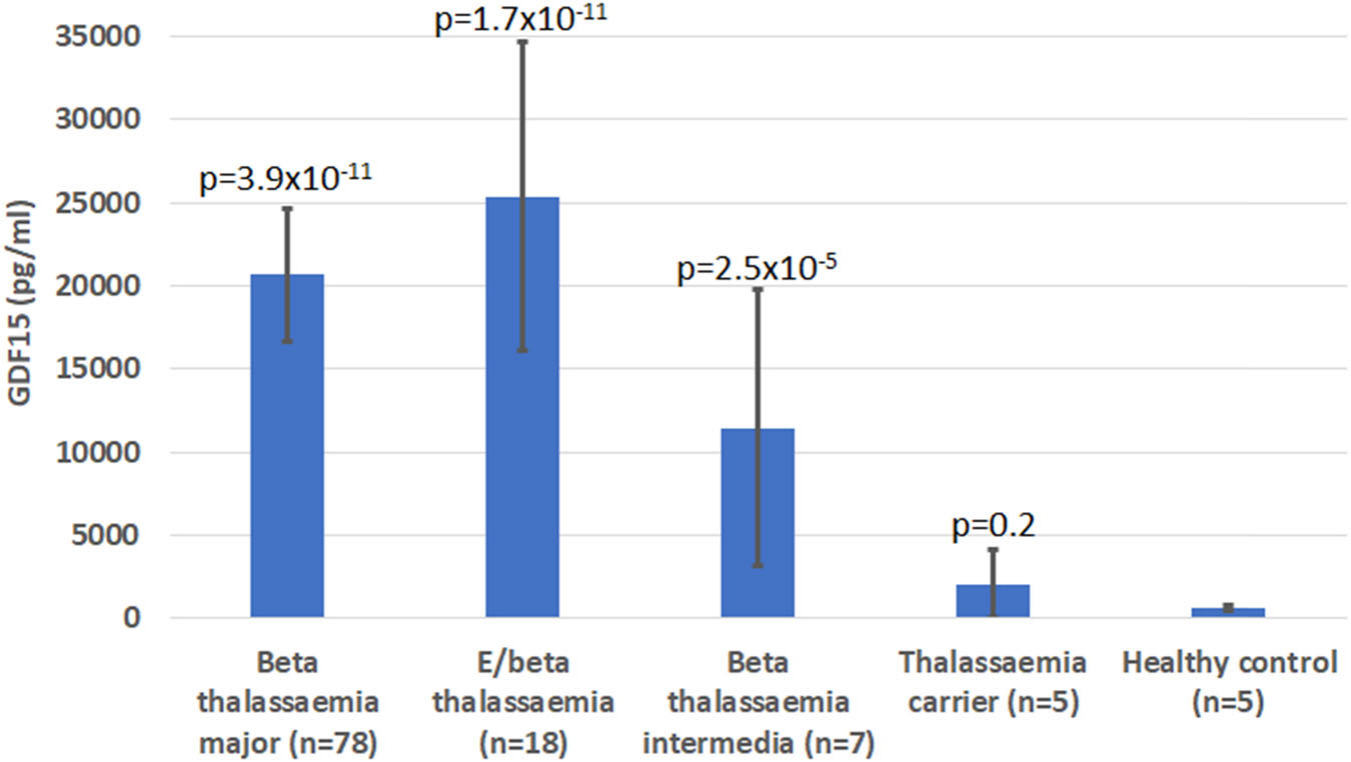
Circulating GDF15 levels in study participants with different haematological diagnoses. Data are shown as geometric means (95% confidence intervals). *p* Values relate to comparisons with healthy controls. [Color figure can be viewed at wileyonlinelibrary.com]

The levels of GDF15 were substantially elevated in patients with β‐thalassaemia when compared to non‐thalassaemia controls (*p* = 3.9 × 10^−11^) and heterozygous carriers of β‐thalassaemia trait (*p* = 1.4 × 10^−7^). This elevation was consistently observed in all subtypes of β‐thalassaemia: β‐thalassaemia major, HbE β‐thalassaemia and β‐thalassaemia intermedia (Figure 1). There was no significant difference of GDF15 concentrations between the three thalassaemia subtypes (Supporting Information: table 2).

Among patients with β‐thalassaemia, GDF15 concentrations were inversely associated with mean pretransfusion haemoglobin levels (standardized *β* −.17 [−.31 to −.03]; *p* = .02). Also, in all participants, GDF15 concentrations were higher in males than in females (*p* = 1.2 × 10^−3^) but were unrelated to age (association with sex after adjustment for age, *p* = 2.1 × 10^−3^, Supporting Information: Figure 1). There was no significant difference between the GDF15 levels in splenectomized (mean 17,654 ± SD 12,490 pg/mL, *n* = 22) and non‐splenectomized (mean 21,737 ± SD 19,112 pg/mL, *n* = 81) patients (*p* = .34). Also, there was no association between the annual blood transfusion volume and GDF15 levels among all patients with thalassaemia (*r* = .008, *p* = .93) (Supporting Information: Figure 2).

### 3.3 Association between GDF15 and BMI

**Figure 2:**
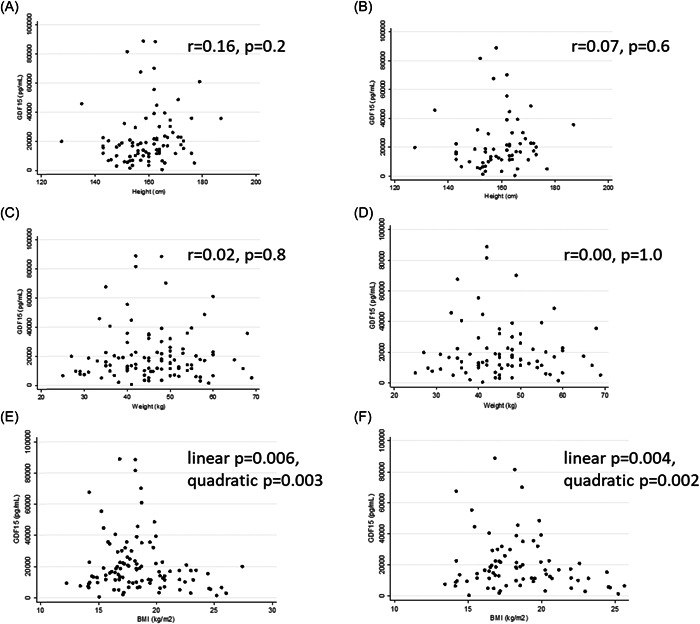
Scatter diagrams showing relationships between circulating GDF15 concentrations and height in (A) all adult participants (*n* = 83) and in (B) adult participants with beta thalassaemia major (*n* = 62), weight in (C) all participants (*n* = 103) and in (D) participants with beta thalassaemia major (*n* = 78)), and BMI in (E) all participants (*n* = 103) and in (F) participants with beta thalassaemia major (*n* = 78). BMI, body mass index.

**Figure 3:**
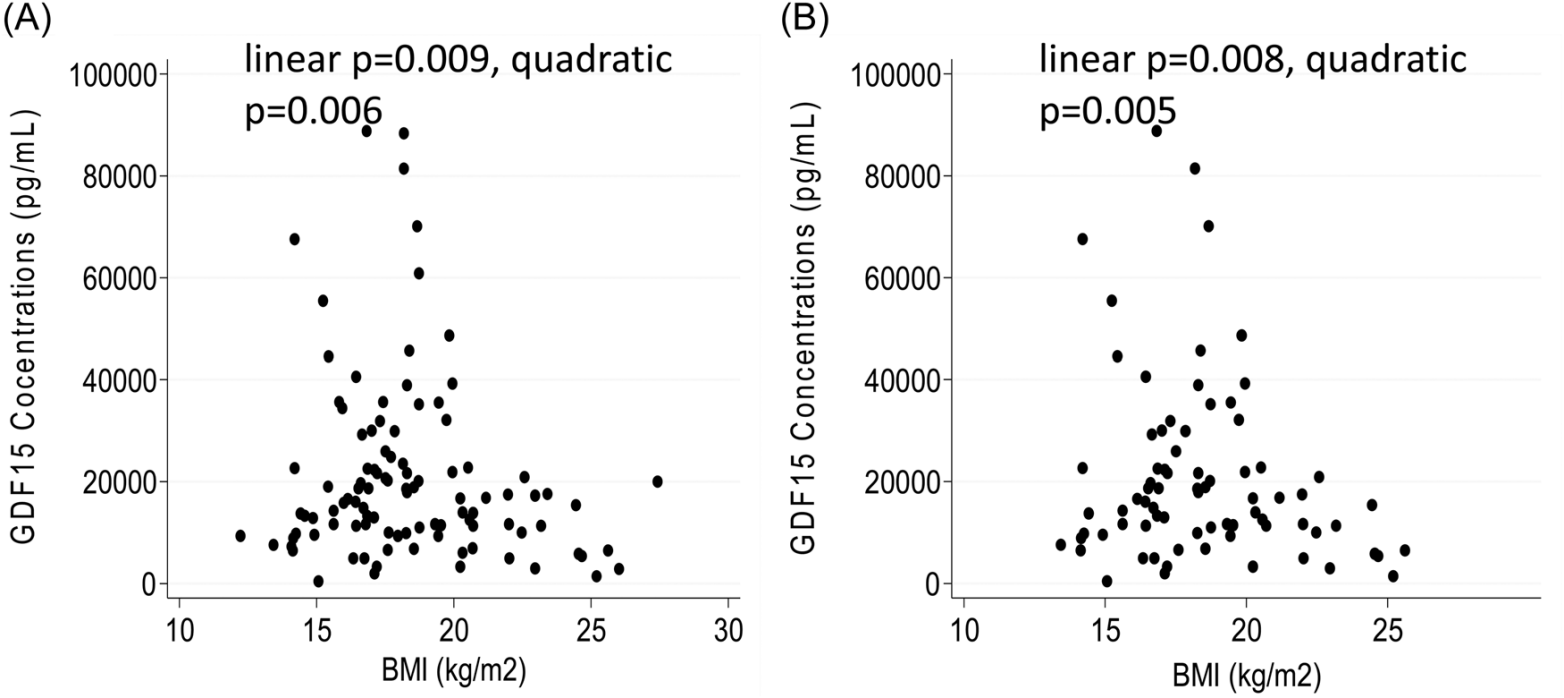
Scatter diagrams showing relationships between natural log‐transformed GDF15 concentrations and BMI in (A) all study participants (*n* = 103) and (B) in participants with beta thalassaemia major (*n* = 78). BMI, body mass index.

Next, we analysed for the associations of GDF15 concentration with anthropometric, appetitive and endocrine markers, both for the All Thalassaemias group [Thal major, E‐Thal and Thal intermedia (*n* = 103)] and for the Thalassaemia Major alone (*n* = 78). In adult participants (age >17 years), after adjustment for age and sex, there was no association between GDF15 and height (Figure 2A,B) or weight (Figure 2C,D) in either the All Thalassaemia or the Thalassaemia Major groups. However, in quadratic models adjusted for sex and age, GDF15 concentrations were associated with BMI in both the All Thalassaemia and the Thalassaemia Major groups (Figure 3). Notably, at levels of GDF15 above 24,000 pg/mL no participant had a BMI above 20 kg/m^2^ and all patients with a BMI > 21 kg/m^2^ had GDF15 levels less than 22,000 pg/mL.

### 3.4 Association between GDF15 and hormone levels

**Figure 4:**
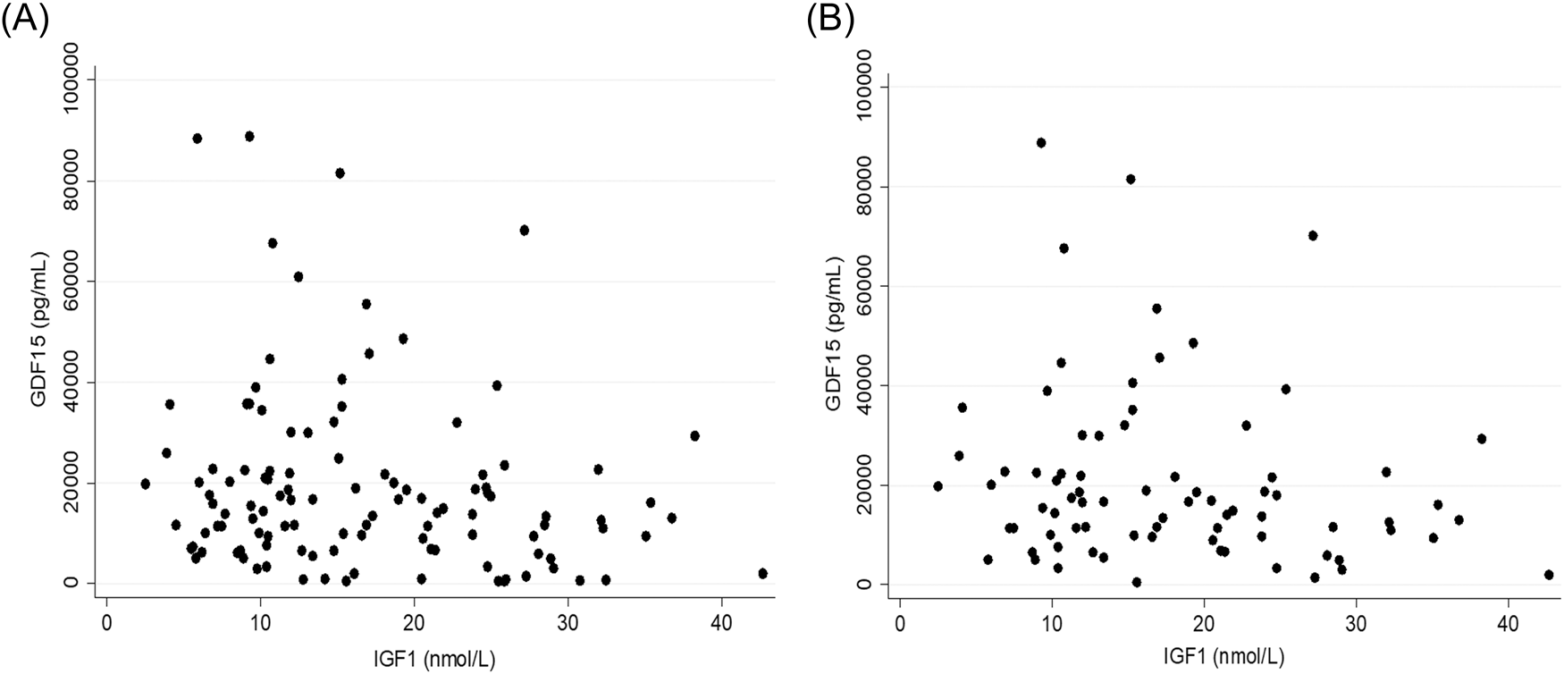
Scatter diagrams showing relationships between circulating IGF1 and GDF15 concentrations in (A) all study participants (*n* = 103) and (B) in participants with beta thalassaemia major (*n* = 78).

IGF1 concentration showed significant inverse correlations with GDF15 concentration (adjusted for BMI, age, sex and Tanner stage) in both the All Thalassaemia and the Thalassaemia Major groups (Figure 4).

GDF15 levels in those treated with thyroxine were 19,983 (11,068−360,810) pg/mL (*n* = 14) and in those untreated 10733 (8602−13,392) pg/mL (*n* = 99) (*p* = .054). GDF15 levels in males treated with testosterone were 18,063 (9648−33,816) pg/mL (*n* = 13) and in those not treated 15,597 (11,292−21,543) pg/mL (*n* = 49) (*p* = .7), and in females treated with oestradiol 15,007 (5789−38,902) pg/mL (*n* = 6) and in those not treated with oestradiol 7182 (5072−10,169) pg/mL (*n* = 45) (*p* = .2). Participants who were not on treatment with thyroxine, testosterone or oestradiol had normal hormone levels and did not require treatment. However, levels of LH, FSH and testosterone (but not oestradiol) were all positively correlated with GDF15 with the relationships being significant after adjusting for age and sex (Supporting Information: Figure 1 and Supporting Information: Tables 3 and 4).

## 4 DISCUSSION

In this study we confirmed previous reports that the β‐thalassaemias are associated with a substantial elevation in levels of circulating GDF15 and that, in patients with β‐thalassaemia major, pretransfusion haemoglobin levels are inversely associated with circulating GDF15. The reason for the higher levels of GDF15 in males than females is unclear.

GDF15 is known to be expressed at high levels in erythroblasts^6^ and its expression is highly responsive to a range of cellular stresses^5^ several of which are likely to be active in the thalassaemic erythroblast undergoing ineffective erythropoiesis. It has been assumed that the extremely high levels of circulating GDF15 in thalassaemia major are derived largely from the haematopoietic compartment but this has not been formally established. All cells can increase GDF15 secretion when stressed and it is possible that non haematopoietic compartments make some contribution to the high GDF15 levels in thalassaemia, which to date has been reported to be a marker of ineffective erythropoiesis and an indicator of disease severity.^14^ The suggestion that it might play a more direct role in the regulation of iron homoeostasis through actions in the liver^6^ are difficult to reconcile with the fact that the only known receptor for GDF15 is exclusively expressed in the brain.

Our study population consisted of patients with transfusion dependent β‐thalassaemia major, HbE β‐thalassaemia and β‐thalassaemia intermedia. Although, HbE β‐thalassaemia is considered a less severe phenotype, our results showed that these patients have the highest GDF15 levels. This could be because patients with HbE β‐thalassaemia are frequently suboptimally transfused due to the lack of uniform guidelines to guide transfusion therapy in these patients.^15^ We recently reported that patients with HbE β‐thalassaemia are chronically under‐transfused and have lower pretransfusion haemoglobin levels and higher prevalence of hepatosplenomegaly compared to patients with β‐thalassaemia major.^16^

The β‐thalassaemias are frequently accompanied by a range of systemic disorders^17^, ^18^ not all of which are necessarily directly related to a low oxygen carrying capacity.^19^ These include failure to thrive, impaired linear growth,^20^ low height, reduced appetite, low body weight and a range of neuroendocrine disorders^21^ including hypothyroidism and central hypogonadism.^22^, ^23^ Iron overload undoubtedly contributes to at least some of these systemic manifestations but despite regular transfusion and iron chelation therapy the prevalence of such disorders remains high.^15^ This led us to speculate whether the chronically high levels of GDF15 might be playing a contributory role in the systemic manifestations of β‐thalassaemia. A definitive test of this hypothesis will require the pharmacological blockade of GDF15 with several relevant agents now in human clinical trials. While awaiting those studies we wished to determine whether there were any demonstrably robust correlations between GDF15 levels and one or more systemic manifestations. We did not register an association between the annual blood transfusion volume and GDF15 levels among all patients with thalassaemia. Similarly, a study done among a cohort of gastric cancer patients did not demonstrate any association between blood transfusion and circulating GDF15 levels.^24^

It has long been established that GDF15 suppresses food intake and body weight in many different species including primates.^25^, ^26^, ^27^ In various disease states, the development of cachexia is strongly associated with the elevation of levels of GDF15.^2^ It is therefore notable that we did see a relationship between GDF15 and BMI in the context of thalassaemia that would be consistent with the known effects of GDF15 to reduce energy stores. The correlations we found were nonlinear, suggesting that there may be some threshold of GDF15 at which effects on energy balance begin to become more marked. Certainly, none of the patients who had a BMI above 21 kg/m^2^ had a GDF15 level that was above 21,000 pg/mL.

There is only limited data on the food intake behaviour and appetite in thalassaemia. There is evidence that patients with thalassaemia have micronutrient deficiencies related to inadequate intake which could be related to poor appetite.^28^

In addition to its effects on appetite, GDF15 powerfully stimulates the hypothalamic pituitary adrenal axis.^29^ No significant correlation was seen with cortisol levels in this study, but the dynamic and circadian nature of cortisol secretion is very poorly captured by random plasma samples, so it is perhaps unsurprising that no correlation was discernible.

Central hypogonadism is one of the commonest endocrine complications of β‐thalassaemia.^30^ We found no differences in GDF15 concentration between patients receiving replacement therapy with gonadal steroids and those not receiving such therapy. This might be due to the fact that hormonal treatment in thalassaemia is rather complex due to the severity of iron overload associated with further endocrine complications^31^ such as accumulation in different endocrine glands.^32^ We noted a surprising, positive relationship between GDF15 levels and LH, FSH and testosterone in males (but not oestradiol in females) each of which remained statistically significant after correction for age, BMI (and sex in the case of LH and FSH). The effects of GDF15 on the hypothalamic pituitary gonadal axis have not been systematically studied in any detail and these findings should prompt further studies.

Disruption of thyroid function is a common feature of Thalassaemia.^33^ Although GDF15 levels were somewhat higher in patients treated with thyroxin than those who were not, this did not reach formal statistical significance. There was no correlation between GDF15 and free T4 or TSH in patients not treated with thyroxine.

The one endocrine marker which was significantly associated with GDF15 was serum IGF1, where the relationship was inverse. IGF1 is profoundly influenced by nutritional state^34^, ^35^ and although this relationship persisted after correction for BMI in our study, it is possible that the association is driven by some unmeasured aspects of undernutrition. Alternatively, glucocorticoid excess suppresses growth hormone and IGF1,^36^ so it is possible that GDF15 might be exerting its effects on IGF1 through its known effects on the hypothalamic pituitary adrenal axis.^29^

In summary, we have conducted the most extensive survey to date of the relationship between the elevated levels of GDF15, which are a consistent feature of the β‐thalassaemias and the systemic complications of this important blood disorder. Our results suggest that GDF15 may play a role in influencing the low adiposity and low IGF1 seen in thalassaemia. However, the definitive test of this hypothesis will require pharmacological studies with antagonists of GDF15 action.

## CONFLICTS OF INTEREST STATEMENT

N. S. has consulted for and/or received speaker honoraria from Abbott Laboratories, Afimmune, Amgen, AstraZeneca, Boehringer Ingelheim, Eli Lilly, Hanmi Pharmaceuticals, Janssen, Merck Sharp & Dohme, Novartis, Novo Nordisk, Pfizer, Roche Diagnostics and Sanofi; and received grant support paid to his University from AstraZeneca, Boehringer Ingelheim, Novartis and Roche Diagnostics outside the submitted work. The remaining authors declare no conflict of interest.

## Supporting information

Supporting information.

## Data Availability

The data sets generated and/or analysed during the current study are not publicly available, since they are subject to national data protection laws and restrictions imposed by the ethics committee to ensure data privacy of the study participants. However, they can be applied for through an individual project agreement with the principal investigators.

## References

[1] Patel S , Alvarez‐Guaita A , Melvin A , et al. GDF15 provides an endocrine signal of nutritional stress in mice and humans. Cell Metab. 2019;29(3):707‐718. 30639358 10.1016/j.cmet.2018.12.016 PMC6408327

[2] Tsai VWW , Lin S , Brown DA , Salis A , Breit SN . Anorexia‐cachexia and obesity treatment may be two sides of the same coin: role of the TGF‐b superfamily cytokine MIC‐1/GDF15. Int J Obes. 2016;40(2):193‐197. 10.1038/ijo.2015.242 26620888

[3] Kempf T , von Haehling S , Peter T , et al. Prognostic utility of growth differentiation factor‐15 in patients with chronic heart failure. JACC. 2007;50(11):1054‐1060. 17825714 10.1016/j.jacc.2007.04.091

[4] Corre J , Hébraud B , Bourin P . Concise review: growth differentiation factor 15 in pathology: a clinical role? Stem Cells Transl Med. 2013;2(12):946‐952. 24191265 10.5966/sctm.2013-0055 PMC3841089

[5] Lockhart SM , Saudek V , O'Rahilly S . GDF15: a hormone conveying somatic distress to the brain. Endocr Rev. 2020;41(4):bnaa007. 32310257 10.1210/endrev/bnaa007 PMC7299427

[6] Tanno T , Bhanu NV , Oneal PA , et al. High levels of GDF15 in thalassemia suppress expression of the iron regulatory protein hepcidin. Nature Med. 2007;13(9):1096‐1101. 17721544 10.1038/nm1629

[7] Tantawy AA , Adly AA , Ismail EA , Darwish YW , Ali Zedan M . Growth differentiation factor‐15 in young sickle cell disease patients: relation to hemolysis, iron overload and vascular complications. Blood Cells Mol Dis. 2014;53(4):189‐193. 25065856 10.1016/j.bcmd.2014.07.003

[8] Tamary H , Shalev H , Perez‐Avraham G , et al. Elevated growth differentiation factor 15 expression in patients with congenital dyserythropoietic anemia type I. Blood. 2008;112(13):5241‐5244. 18824595 10.1182/blood-2008-06-165738 PMC2954708

[9] Klein AB , Nicolaisen TS , Ørtenblad N , et al. Pharmacological but not physiological GDF15 suppresses feeding and the motivation to exercise. Nat Commun. 2021;12(1):1041. 33589633 10.1038/s41467-021-21309-x PMC7884842

[10] Lakhal S , Talbot NP , Crosby A , et al. Regulation of growth differentiation factor 15 expression by intracellular iron. Blood. 2009;113(7):1555‐1563. 19047680 10.1182/blood-2008-07-170431

[11] Taher AT , Musallam KM , Cappellini MD . β‐thalassemias. N Engl J Med. 2021;384(8):727‐743. 33626255 10.1056/NEJMra2021838

[12] Premawardhana AP , Mudiyanse R , De Silva ST , et al. A nationwide survey of hospital‐based thalassemia patients and standards of care and a preliminary assessment of the national prevention program in Sri Lanka. PLoS One. 2019;14(8):e0220852. 31419232 10.1371/journal.pone.0220852 PMC6697367

[13] Mettananda S , Pathiraja H , Peiris R , et al. Health related quality of life among children with transfusion dependent β‐thalassaemia major and haemoglobin E β‐thalassaemia in Sri Lanka: a case control study. Health Qual Life Outcomes. 2019;17(1):137. 31395066 10.1186/s12955-019-1207-9 PMC6686351

[14] Musallam KM , Taher AT , Duca L , Cesaretti C , Halawi R , Cappellini MD . Levels of growth differentiation factor‐15 are high and correlate with clinical severity in transfusion‐independent patients with β thalassemia intermedia. Blood Cells Mol Dis. 2011;47(4):232‐234. 21865063 10.1016/j.bcmd.2011.07.005

[15] Premawardhena AP , Ediriweera DS , Sabouhanian A , et al. Survival and complications in patients with haemoglobin E thalassaemia in Sri Lanka: a prospective, longitudinal cohort study. Lancet Global Health. 2022;10(1):e134‐e141. 34843671 10.1016/S2214-109X(21)00446-0 PMC8672061

[16] Mettananda S , Pathiraja H , Peiris R , et al. Blood transfusion therapy for beta‐thalassemia major and hemoglobin E beta‐thalassemia: adequacy, trends, and determinants in Sri Lanka. Pediatr Blood Cancer. 2019;66(5):e27643. 30697927 10.1002/pbc.27643

[17] Piriyakhuntorn P , Tantiworawit A , Kasitanon N , Louthrenoo W . Prevalence and characteristics of inflammatory rheumatic diseases in patients with thalassemia. Ann Hematol. 2022;101(8):1667‐1675. 35604471 10.1007/s00277-022-04870-3

[18] Noureldine MHA , Taher AT , Haydar AA , Berjawi A , Khamashta MA , Uthman I . Rheumatological complications of beta‐thalassaemia: an overview. Rheumatology. 2018;57(1):19‐27. 28371817 10.1093/rheumatology/kex058

[19] Allen A , Perera S , Mettananda S , et al. Oxidative status in the β‐thalassemia syndromes in Sri Lanka; a cross‐sectional survey. Free Radic Biol Med. 2021;166:337‐347. 33677065 10.1016/j.freeradbiomed.2021.02.028

[20] Scacchi M , Danesi L , Cattaneo A , et al. Growth hormone deficiency (GHD) in adult thalassaemic patients. Clin Endocrinol. 2007;67(5):790‐795. 10.1111/j.1365-2265.2007.02965.x 17608814

[21] Di Maio S , Marzuillo P , Mariannis D , et al. A retrospective long‐term study on age at menarche and menstrual characteristics in 85 young women with transfusion‐dependent β‐thalassemia (TDT). Mediterr J Hematol Infect Dis. 2021;13(1):e2021040. 34276909 10.4084/MJHID.2021.040 PMC8265331

[22] Taher AT , Weatherall DJ , Cappellini MD . Thalassaemia. Lancet. 2018;391(10116):155‐167. 28774421 10.1016/S0140-6736(17)31822-6

[23] Vidal A , Dhakal C . Association of beta‐thalassaemia and hypogonadotropic hypogonadism. Case Rep Obstet Gynecol. 2022;2022:1‐5. 10.1155/2022/4655249 PMC9135550 35646403

[24] Liu J , Chen S , Ye X . The effect of red blood cell transfusion on plasma hepcidin and growth differentiation factor 15 in gastric cancer patients: a prospective study. Ann Transl Med. 2019;7(18):466. 31700902 10.21037/atm.2019.08.33 PMC6803218

[25] Katsumura S , Siddiqui N , Goldsmith MR , et al. Deadenylase‐dependent mRNA decay of GDF15 and FGF21 orchestrates food intake and energy expenditure. Cell Metab. 2022;34(4):564‐580. 35385705 10.1016/j.cmet.2022.03.005 PMC9386786

[26] Wang D , Day EA , Townsend LK , Djordjevic D , Jørgensen SB , Steinberg GR . GDF15: emerging biology and therapeutic applications for obesity and cardiometabolic disease. Nat Rev Endocrinol. 2021;17(10):592‐607. 34381196 10.1038/s41574-021-00529-7

[27] Day EA , Ford RJ , Smith BK , et al. Metformin‐induced increases in GDF15 are important for suppressing appetite and promoting weight loss. Nature Metabolism. 2019;1(12):1202‐1208. 10.1038/s42255-019-0146-4 32694673

[28] Al‐Naama LM , Hassan MK , Abdul Karim MM . Evaluation of serum leptin levels and growth in patients with β‐thalassaemia major. Anemia. 2016;2016:1‐7. 10.1155/2016/8454286 PMC4819093 27088012

[29] Cimino I , Kim H , Tung YCL , et al. Activation of the hypothalamic‐pituitary‐adrenal axis by exogenous and endogenous GDF15. Proc Natl Acad Sci. 2021;118(27):e2106868118. 34187898 10.1073/pnas.2106868118 PMC8271778

[30] Higgs DR , Engel JD , Stamatoyannopoulos G . Thalassaemia. Lancet. 2012;379(9813):373‐383. 21908035 10.1016/S0140-6736(11)60283-3

[31] De Sanctis V , Soliman AT , Daar S , Di Maio S . Adverse events during testosterone replacement therapy in 95 young hypogonadal thalassemic men. Acta bio‐medica: Atenei Parmensis. 2019;90(2):228‐232. 31125000 10.23750/abm.v90i2.8477 PMC6776204

[32] Hagag AA , Badraia IM , Elfarargy MS , Abo El‐Enein AM . Gonadal hormones in adolescent females with β‐thalassemia in relation to iron load. Endocr Metab Immune Disord Drug Targets. 2016;16(2):148‐153. 27150601 10.2174/1871530316666160506150516

[33] Bordbar M , Bozorgi H , Saki F , et al. Prevalence of endocrine disorders and their associated factors in transfusion‐dependent thalassemia patients: a historical cohort study in Southern Iran. J Endocrinol Invest. 2019;42(12):1467‐1476. 31228105 10.1007/s40618-019-01072-z

[34] Estívariz CF , Ziegler TR . Nutrition and the insulin‐like growth factor system. Endocrine. 1997;7(1):65‐71. 9449035 10.1007/BF02778066

[35] Hawkes CP , Grimberg A . Insulin‐like growth factor‐I is a marker for the nutritional state. Pediatric Endocrinol Rev: PER. 2015;13(2):499‐511. PMC5576178 26841638

[36] Morgan SA , Berryman DE , List EO , Lavery GG , Stewart PM , Kopchick JJ . Regulation of 11β‐HSD1 by GH/IGF‐1 in key metabolic tissues may contribute to metabolic disease in GH deficient patients. Growth Hormone IGF Res. 2022;62:101440. 10.1016/j.ghir.2021.101440 34814007

