# Supplementary material for: Association of GDF15 levels with body mass index and endocrine status in β‐thalassaemia
Source: Clin Endocrinol (Oxf). 2023 Feb 27;99(2):182–9. doi: 10.1111/cen.14897 (PMC10952638; doi:10.1111/cen.14897)
Supplement: Supplementary file 1 — Supporting information. [file CEN-99-182-s001.docx]

**Legends for supplementary tables and figures**

Supplementary Table 1. Non-specific symptoms related to food intake and lifestyle among patients with β-thalassaemia

Supplementary Table 2. GDF15 concentrations (pg/mL) in different subtypes of β-thalassaemia

Supplementary Table 3. Associations between circulating GDF15 concentrations and poor appetite classification and hormone concentrations in all participants with β-thalassaemia.

Supplementary Table 4. Associations between circulating GDF15 concentrations and poor appetite classification and hormone concentrations in participants with β-thalassaemia major.

Supplementary Figure 1. Scatter diagrams showing relationships between circulating GDF15 concentrations and (a) TSH (n=89, patients treated with thyroxine excluded), (b) free thyroxine (n=83, patients treated with thyroxine excluded), (c) cortisol (n=99), (d) FSH (n=99, one participant with a FSH of 81 IU/mL excluded from scatter diagram, patients treated with testosterone or estriol excluded), (e ) LH (n=99, patients treated with testosterone or estriol excluded), (f) testosterone (n=62, males only, patients treated with testosterone excluded) and (g) oestradiol (n=51, females only, patients treated with estriol excluded) in all participants.

Supplementary Figure 2. Scatter diagram showing the relationship between the annual blood transfusion requirement and GDF15 concentrations.

**Supplemental data**

**Supplementary Table 1.** Prevalence of non-specific symptoms related to food intake and lifestyle among patients with thalassaemia

| **Symptom** | **Overall**  **(N=103)** | **When haemoglobin is low**  **(Before a routine blood transfusion) (N=103)** |
| --- | --- | --- |
| Poor appetite | 5 (4.9%) | 37 (35.9%) |
| Nausea | 3 (2.9%) | 2 (1.9%) |
| Abdominal discomfort | 4 (3.9%) | 3 (2.9%) |
| Fatigue | 2 (1.9%) | 89 (86.4%) |
| Sleepiness | 2 (1.9%) | 68 (66.0%) |
| Backache | 10 (9.7%) | 33 (32.0%) |

**Supplementary Table 2.** GDF15 concentrations (pg/mL) in different subtypes of β-thalassaemia

|  | **β-thalassaemia major**  **(N=78)** | **HbE β-thalassaemia**  **(N=18)** | **β-thalassaemia intermedia**  **(N=7)** | **β-thalassaemia trait**  **(N=5)** | **Non-thalassaemia controls**  **(N=5)** |
| --- | --- | --- | --- | --- | --- |
| Mean | 20668 | 25375 | 11458 | 1993 | 619 |
| Geometric mean | 14532 | 20944 | 8609 | 1215 | 602 |
| SD | 17786 | 19863 | 11045 | 2407 | 157 |
| Minimum | 400 | 9306 | 2847 | 407 | 413 |
| Maximum | 88760 | 88360 | 35685 | 6184 | 814 |
| Difference in mean compared to non-thalassaemia controls | p=3.9x10^-11^ | p=1.7x10^-11^ | p=2.5x10^-5^ | p=0.2 | – |
| Difference in mean compared to thalassaemia major | – | 0.1 | 0.1 | P=1.4x10^-7^ | p=3.9x10^-11^ |

| **Characteristic** | **Unadjusted** | | | **Adjusted for sex and BMI** | | |
| --- | --- | --- | --- | --- | --- | --- |
|  | Standardised β | p-value | n | Standardised β | p-value | n |
| Poor Appetite Classification | -0.020  (-0.193, 0.154) | 0.8 | 103 | -0.023  (-0.186, 0.140) | 0.8 | 103 |
| Cortisol | -0.101  (-0.273, 0.071) | 0.2 | 99 | -0.077  (-0.240, 0.086) | 0.3 | 99 |
| Free Thyroxine  (excluding those treated with thyroxine) | -0.076  (-0.237, 0.085) | 0.4 | 83 | -0.042  (-0.201, 0.118) | 0.6 | 83 |
| FSH  (excluding those treated with testosterone or oestradiol) | 0.124  (-0.072, 0.319) | 0.2 | 74 | 0.184  (-0.005, 0.374) | 0.06 | 74 |
| LH  (excluding those treated with testosterone or oestradiol) | 0.266  (0.076, 0.457) | **0.007** | 74 | 0.272  (0.088, 0.455) | **0.004** | 74 |
| Oestradiol  (females only, excluding those treated with oestradiol) | -0.132  (-0.447, 0.183) | 0.4 | 45 | -0.038  (-0.254, 0.179) | 0.7 | 38 |
| Testosterone  (males only, excluding those treated with testosterone) | 0.278  (0.046, 0.510) | **0.02** | 62 | 0.233  (0.061, 0.405) | **0.009** | 59 |
| TSH  (excluding those treated with thyroxine) | -0.027  (-0.201, 0.146) | 0.8 | 89 | -0.013  (-0.179, 0.153) | 0.9 | 89 |

**Supplementary Table 3.** Associations between circulating GDF15 concentrations and poor appetite classification and hormone concentrations in all participants with β-thalassaemia.

**Supplementary Table 4.** Associations between circulating GDF15 concentrations and poor appetite classification and hormone concentrations in participants with β-thalassaemia major.

| **Characteristic** | **Unadjusted** | | | **Adjusted for sex and BMI** | | |
| --- | --- | --- | --- | --- | --- | --- |
|  | Standardised β | p-value | n | Standardised β | p-value | n |
| Poor Appetite Classification | -0.010  (-0.182, 0.162) | 0.9 | 78 | -0.023  (-0.182, 0.136) | 0.8 | 78 |
| Cortisol | -0.014  (-0.200, 0.171) | 0.9 | 76 | 0  (-0.176, 0.175) | 1.0 | 76 |
| Free Thyroxine  (excluding those treated with thyroxine) | -0.077  (-0.264, 0.109) | 0.4 | 64 | -0.025  (-0.212, 0.162) | 0.8 | 64 |
| FSH  (excluding those treated with testosterone or oestradiol) | 0.229  (-0.050, 0.508) | 0.1 | 54 | 0.363  (0.090, 0.636) | **0.01** | 54 |
| LH  (excluding those treated with testosterone or oestradiol) | 0.296  (0.059, 0.533) | **0.02** | 54 | 0.341  (0.108, 0.573) | **0.005** | 54 |
| Oestradiol  (females only, excluding those treated with oestradiol) | -0.147  (-0.433, 0.140) | 0.3 | 26 | -0.036  (-0.315, 0.243) | 0.8 | 26 |
| Testosterone  (males only, excluding those treated with testosterone) | 0.286  (0.098, 0.474) | **0.004** | 49 | 0.218  (0.032, 0.405) | **0.02** | 49 |
| TSH  (excluding those treated with thyroxine) | -0.036  (-0.233, 0.162) | 0.7 | 68 | -0.045  (-0.234, 0.145) | 0.6 | 68 |

**Supplementary Figure 1.**


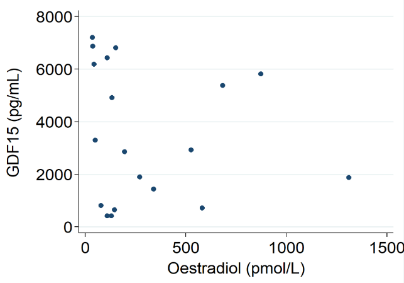

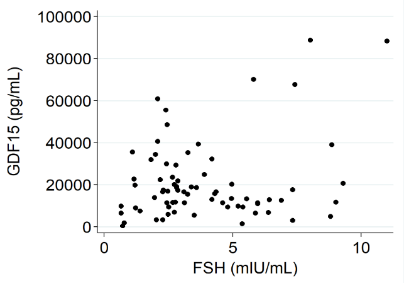

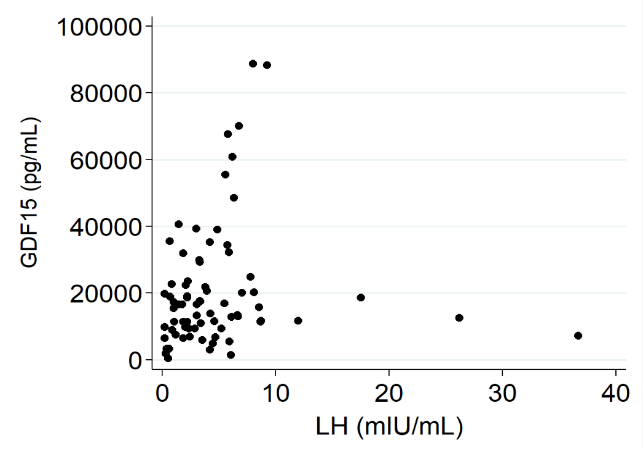

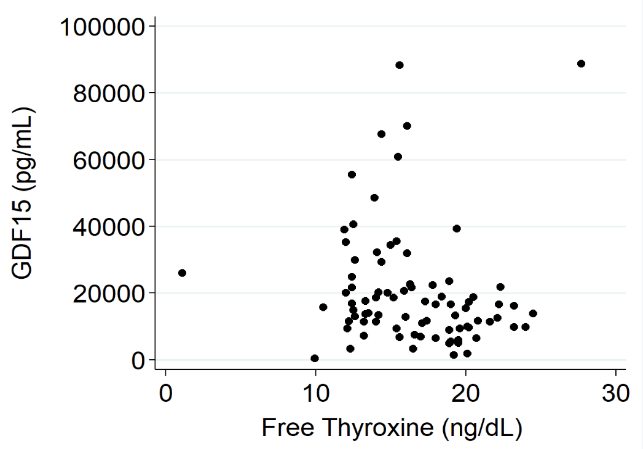

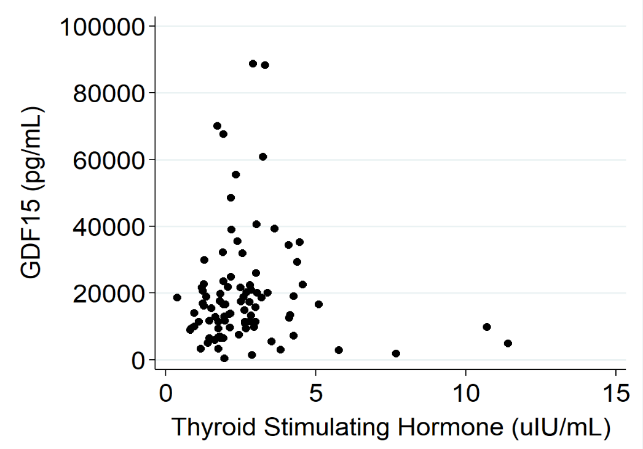

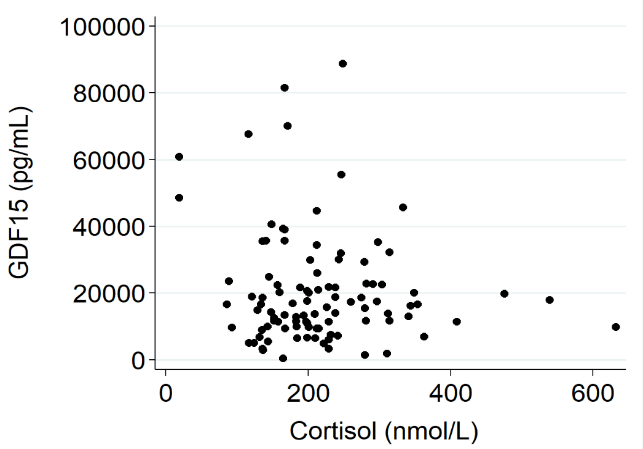

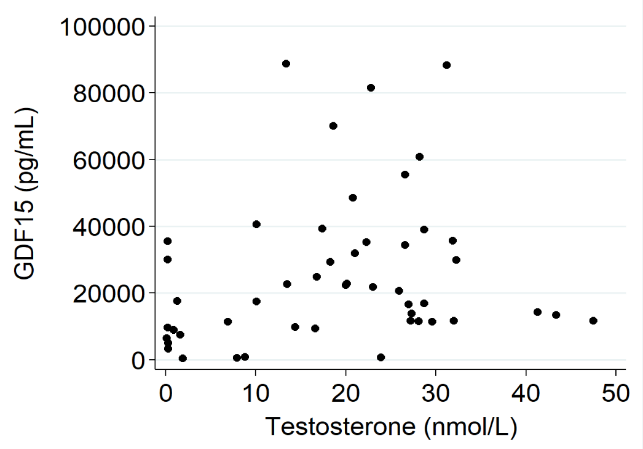


**A**

**r=-0.03, p=0.8**

**B**

**r=-0.10, p=0.4**

**C**

**r=-0.04, p=0.2**

**D**

**r=0.15, p=0.2**

**E**

**r=0.31, p=0.007**

**F**

**r=0.26, p=0.02**

**G**

**r=-0.13, p=0.4**

**Supplementary Figure 2.**


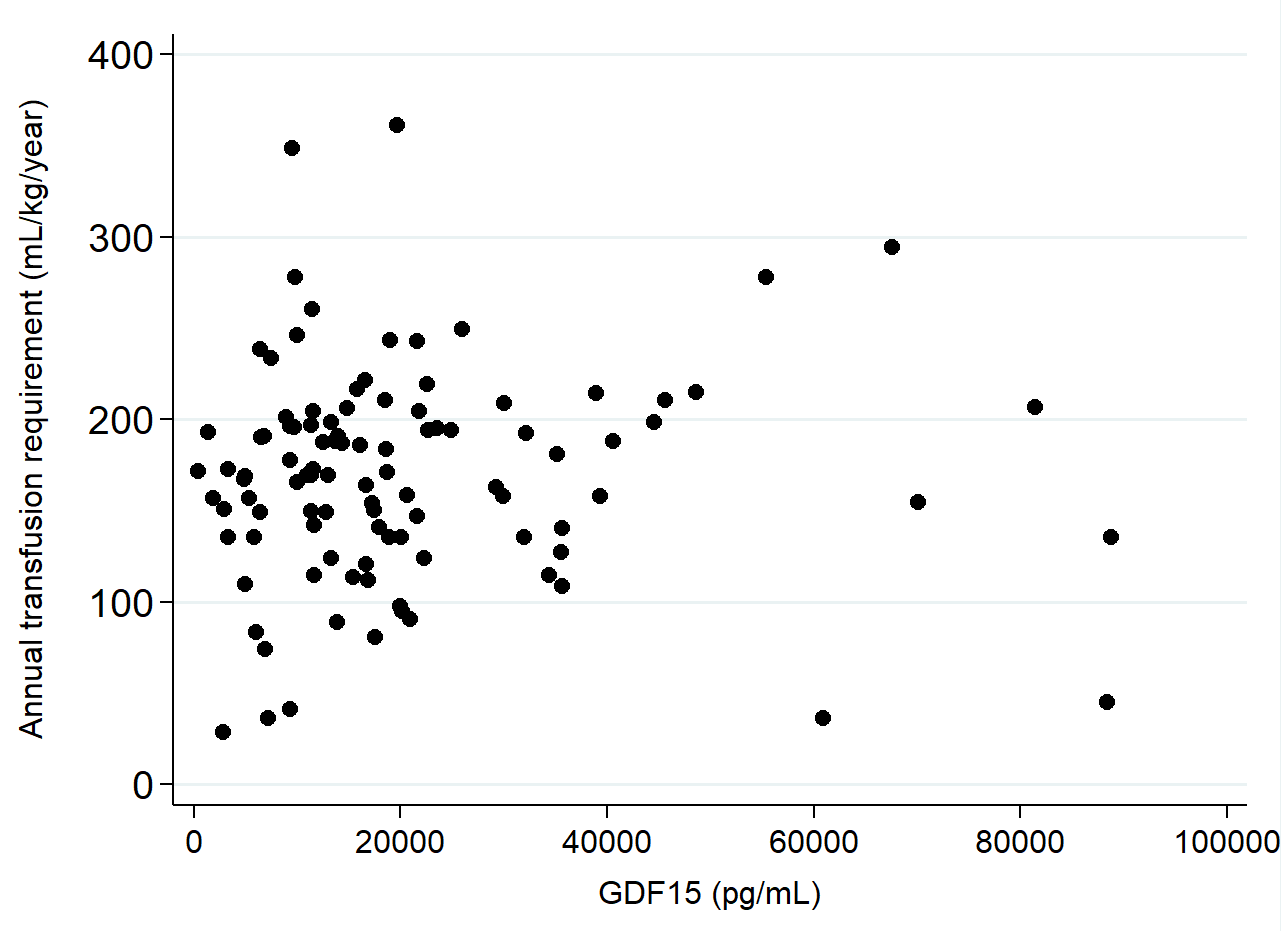


**r=0.008, p=0.93**
